# Do Sex/Gender and Menopause Influence the Psychopathology and Comorbidity Observed in Delusional Disorders?

**DOI:** 10.3390/jcm11154550

**Published:** 2022-08-04

**Authors:** Alexandre González-Rodríguez, Mary V. Seeman, Alexandre Díaz-Pons, Rosa Ayesa-Arriola, Mentxu Natividad, Eva Calvo, José A. Monreal

**Affiliations:** 1 Department of Mental Health, Mutua Terrassa University Hospital, Fundació Docència i Recerca Mutua Terrassa, University of Barcelona (UB), Centro de Investigación Biomédica en Red de Salud Mental (CIBERSAM), 08221 Terrassa, Spain; 2 Department of Psychiatry, University of Toronto, Toronto, ON M5P 3L6, Canada; 3 Department of Psychiatry, Marqués de Valdecilla University Hospital, IDIVAL, School of Medicine, University of Cantabria, 39008 Santander, Spain; 4 Faculty of Psychology, National University of Distance Education (UNED), Centro de Investigación Biomédica en Red de Salud Mental (CIBERSAM), 28029 Madrid, Spain; 5 Institut de Neurociències, Universitat Autònoma de Barcelona, 08221 Terrassa, Spain

**Keywords:** delusional disorders, psychosis, sex, women, psychopathology

## Abstract

*Background*: While sex differences and gonadal hormone levels are taken seriously in the understanding and treatment of schizophrenia, their influence in the psychopathology of delusional disorders (DD) remains unknown. *Methods*: Our strategy was to conduct a narrative review of the effects of (a) sex/gender difference and (b) menopause on delusional content, affective and anxiety-related comorbidity, substance use disorders, cognition, aggressivity, and suicide risk in DD. *Results*: Because the literature is scarce, our results are tentative. We found that erotomania was more prevalent in women than in men, and especially in women with premenopausal onset. In contrast, jealous and somatic delusions were more commonly seen in DD women with postmenopausal onset. With respect to depressive comorbidity, women with premenopausal onset appear more vulnerable to depression than those with later onset. Age at menopause is reported to correlate positively with intensity of suicidal ideation. Anxiety symptoms may be related to estrogen levels. Men present with higher rates of substance use disorders, particularly alcohol use. *Conclusions*: Many male/female differences in DD may be attributable to sociocultural factors but menopause, and, therefore, levels of female hormones, influence symptom expression in women and mediate the expression of psychiatric comorbidities. Further research in this area promises to lead to improved individualized treatment.

## 1. Introduction

In schizophrenia studies, gender differences in the epidemiology, age of onset, psychopathology, and clinical course have been extensively reported [1]. In fact, one of the most stable findings in psychiatry research is that women with schizophrenia show a later age of onset than men, and that, while the peak of incidence in both sexes occurs in late adolescence and young adulthood, women experience a second peak at the end of their reproductive life [2]. In addition, epidemiological studies indicate that the incidence of schizophrenia is higher in men than in women [2], and that this demographic difference diminishes when age and menopausal status are controlled [3].

With respect to clinical symptoms in schizophrenia, sex/gender differences are more controversial [2,4]. Many results indicate that men suffer more ‘negative’ symptoms (apathy, avolition, anhedonia, and social withdrawal) while women suffer more affective symptoms (depression and mood swings) [2], but recent studies have pointed to the confounding effects on symptoms of several associated factors. A study carried out by Riecher-Rössler and colleagues [5] assessed psychopathological symptoms in 117 individuals diagnosed with an at-risk mental state (ARMS) for psychosis and 87 first-episode of psychosis (FEP) patients. No sex/gender differences in psychopathology, as measured by self-report or observer rated scales, were found. These findings are in agreement with more recent results [4] comparing psychosis patients with healthy controls.

The same is true for research that points to sex/gender differences in antipsychotic response and clinical outcomes in schizophrenia [6]. The findings are that women, in general, show a stronger response to antipsychotic medications than men during the reproductive years, but that this is no longer the case after menopause [7,8,9]. Response appears to worsen in women with time after menopause, suggesting that the further decline of estrogen at adrenopause contributes to the loss of effective antipsychotic response [10].

The relevance of a hormonal effect is reinforced by the success of raloxifene, a selective estrogen receptor modulator, in reducing the severity of psychotic symptoms when it is used as an adjunct to antipsychotics [11]. Using pooled data from two previous clinical trials, the Kulkarni group found that 120 mg/day of adjunctive raloxifene over a 12-week period significantly improved cognitive performance over that of placebo. After stratifying for menopausal status and adjusting for endogenous hormone levels (estrogen, progesterone, follicle stimulating hormone, and luteinizing hormone), semantic fluency, picture naming, and word list recognition were all improved by the addition of raloxifene. Aside from showing the effectiveness of hormonal treatment, this study also highlights the importance of considering menopause status when interpreting treatment effects [12].

The effect of male hormones in schizophrenia has been less often considered. A study of 120 male schizophrenia patients found that, in non-aggressive patients, lower levels of testosterone were associated with greater severity of negative symptoms [13], but association in aggressive patients remains unclear [14].

Despite accumulating evidence supporting sex differences attributable to gonadal hormones in schizophrenia, analogous differences in delusional disorders (DD) have been rarely investigated although these disorders have been known and written about since the time of Kraepelin [15] and Bleuler [16].

Several classic syndromes have been associated with DD, such as Othello syndrome [17], delusional jealousy often associated with alcohol and dementing illness and male sex. There is also de Clérambault syndrome [18] an erotomania syndrome associated with young women, and Ekbom syndrome (delusional parasitosis) [19] typically seen in middle aged women.

Because DD and schizophrenia, though related, are distinct disorders that differ in epidemiology, symptoms, and management [20,21], the investigation of DD differences between men and women is indicated. There is, for instance, significantly more functional deterioration in schizophrenia than in DD and this has been attributed to lesser neuropsychological impairment in DD [22]. More recent work, however, reports similar cognitive profiles in the two conditions [23]. While both schizophrenia and DD are characterized by the presence of delusions, in DD they generally less bizarre. DD has been classified in the Diagnostic and Statistical Manual for Mental Disorders, Fifth Edition (DSM-5), into seven subtypes according to the predominant delusional theme: persecutory, erotomanic, jealous, grandiose, somatic, mixed, and unspecified [24]. Most studies report that DD is more frequent (1.2:1–1.6:1) in women than in men; however, some have not been able to replicate these findings [24]. DSM-5 reports no major sex/gender differences in the prevalence of DD [25]. The International Statistical Classification of Diseases and Related Health Problems, 11th Edition (ICD-11) does not address sex/gender demographics but lists persistence as a characteristic of DD and unaffected affect, speech, and behavior as a requirement for diagnosis [26].

For many decades, clinical evidence has suggested that gonadal hormones may be partially responsible for the sex differences that have been found in schizophrenia [27,28]. This is because physiological estrogen fluctuations in women have been observed to affect symptom levels. Estrogens serve many neuroprotective functions, and the observations are that psychotic symptoms in women with schizophrenia wane when estrogen levels are high and rise when they are low [29].

The goal of this review is to explore the literature on the effects of sex/gender on the psychopathology of DD. Specifically, we address the following questions: (1) Are there male/female differences in delusional content in DD? What is the effect of menopause on the sex distribution of delusional themes? (2) Are there gender differences in depressive comorbidity and prevalence of anxiety disorders in patients with DD? What is the effect of menopause on the occurrence and expression of affective and/or anxiety symptoms? (3) Are there gender differences in substance use disorders in DD? What is the effect of menopause on substance use comorbidity? (4) Is suicide and aggressivity risk in DD gender-dependent? What is the effect of menopause on suicidality and aggressivity? (5) Are there gender differences in cognitive symptoms in DD? What is the effect of menopause on cognition?

We use the word ‘sex’ when referring to strictly biological causation of male/female difference and the word ‘gender’ when the differences have sociocultural roots although, in practice, the origin of difference is both biological and sociocultural.

## 2. Methods

A narrative review was conducted based on electronic searches through the PubMed database for English, Spanish, German, or French papers that referred in their titles or abstracts to sex/gender difference, hormones, menopause, or psychopathology in patients with DD. Additionally, we searched for further papers through the Clinicaltrials.gov database. We included papers if they addressed potential hormonal effects as explanations for male/female differences in psychopathological symptoms, psychiatric comorbidity, or suicide risk in patients with DD.

The following keywords were used: (sex OR gender OR hormones OR menopause OR women OR female) AND (“delusional disorder”). The screening and selection process was undertaken by A.G.R. and M.V.S. A total of 489 titles and abstracts were scanned. Most were excluded as they did not address the questions in which we were interested. The inclusion criteria were as follows: (1) randomized controlled trials, or (2) observational and prospective cohort studies, or (3) retrospective studies, as long as (4) they reported potential associations between sex hormones and psychopathological symptoms (including cognition) or psychiatric comorbidity or suicide risk in DD patients. Case reports were excluded.

**Figure 1:**
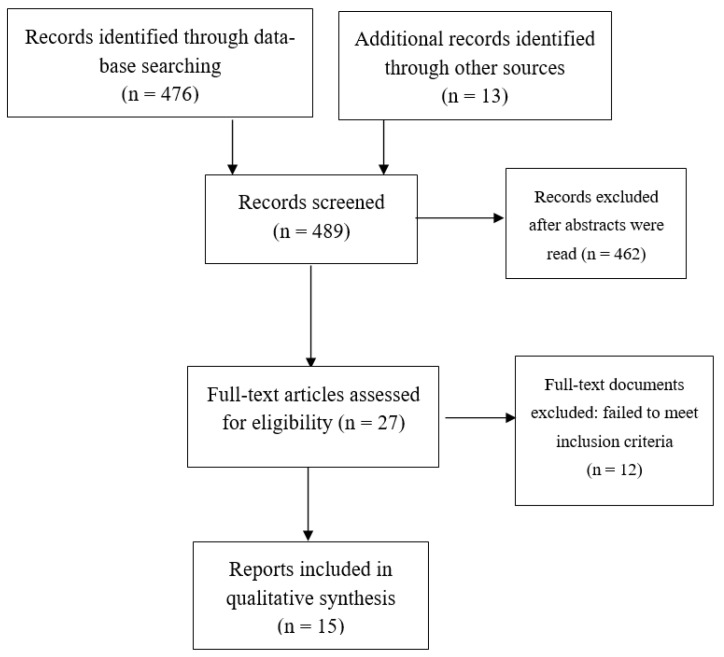
Flow diagram of included studies.

Figure 1 shows the methodological procedure and results of the screening and selection process. After screening all accessible full-text papers, a total of 15 records were identified as relevant to our questions.

The Scale for the Assessment of Narrative Review Articles (SANRA) was used to evaluate the quality of our narrative review [30]. The scale consists of six items rated from zero (low standard) to two (high standard). Item 1 refers to the justification of the article’s importance for the readership. Item 2: Presence of a statement of concrete aims or formulation of questions. Item 3: Description of the literature search. Item 4: Inclusion of references. Item 5: Demonstration of scientific reasoning. Item 6: Appropriate presentation of data. All six items were checked, and the checklist for this review is shown as Table 1.

## 3. Results

### 3.1. The Effects of Sex/Gender on Delusional Themes in Delusional Disorders

Table 2 summarizes findings on the investigation of the correlation between gender and delusional content in DD.

Wustmann and collaborators carried out a gender analysis in a cohort of patients with DD as part of the Halle Delusional Syndrome Study (HADES-Study) [31]. In the first part of this study, 43 consecutive inpatients (22 m; 21 f) who fulfilled either the Diagnostic and Statistical Manual for Mental Disorders, Fourth Edition (DSM-IV) or the International Classification of Diseases 10th Edition (ICD-10) criteria for DD were followed for a minimum of three years and a maximum of 24 years. The men had more history of perinatal disturbances, lower social support and were more frequently single than women. Age at first symptom of DD and age at first hospital admission were higher in women than men, potentially due to the neuroprotective effects of estrogens. No statistically significant differences were found in the thematic content of delusions (i.e., persecutory, somatic, jealous, grandiose, and erotomanic delusions) did not differ in prevalence between men and women. Diagnostic conversion to other psychiatric conditions during the follow-up period was more frequent in men than in women. Women received psychopharmacological treatment more frequently than men. This could mean they were seen as more severely ill or, conversely, more willing to comply with medical directives and more adherent to their medication regimen.

In a similar study, Román-Avezuela and colleagues explored gender differences in a sample of 50 inpatients with DD [32]. Patients (22 m and 28 f) who fulfilled Diagnostic and Statistical Manual for Mental Disorders, 3rd Edition Revised (DSM-III-R), DSM-IV or ICD-10 criteria were consecutively recruited. Women’s hospitalizations occurred at older ages than men’s and women were more likely to suffer from depressive symptoms. Men presented with more persecutory, grandiose, and jealous delusions than women. Erotomania, on the other hand, was more commonly seen in women.

More recently, Kulkarni et al. [33] compared medical records of 455 patients diagnosed with DD (236 m; 219 f) with respect to age, sociodemographics, age at onset and duration of symptoms, family history, clinical and treatment details, and hospitalizations. No gender differences were found regarding age of onset or phenomenology of delusions. However, men were more likely than women to present with delusions of dysmorphophobia. In the overall sample, delusions of jealousy were the most common, followed by persecutory delusions and erotomania. Along the same lines, de Portugal and collaborators explored gender differences in DD in a cross-sectional study of 86 outpatients without finding significant differences regarding delusional content [34]. All participants were screened with the Structured Clinical Interview for the major DSM-IV Axis I diagnoses (SCID-I). Persecution was the most common delusional theme, followed by jealousy and erotomania. Men scored higher than women on symptom severity due to more frequent general and negative symptoms.

An Australian descriptive study investigated antipsychotic use, treatment outcomes, and clinical features in 55 individuals with DD aged 65 and older [35]. The patients were attending a psychiatry service, and the vast majority were postmenopausal women. The mean age at service presentation was 74.5 years, and the average age at onset was 67.5 years. The vast majority presented with persecutory delusions, six with delusional jealousy and one with delusional parasitosis. No gender difference was found with regard to delusional subtype. In another study of a psychogeriatric population, Leinonen and collaborators followed a cohort of 24 patients with major depressive disorders and 18 patients diagnosed with DD [36]. The mean age of the DD group was 75.8 and 89% were women. Five patients developed dementia. The postmenopausal women showed cognitive decline. Consistent with these findings were the results of a case register study of patients aged 60 or older [37] from a catchment area of the southern district of Amsterdam. The one-year prevalence of DD was estimated at 0.03%, and, in women, was found only in those aged 70 years and older.

A prospective study of 43 women with schizophrenia and related disorders (which includes DD) investigated the association between menstrual cycle and hospital admission. The comparison group was 14 women with other psychiatric diagnoses (affective disorder, anxiety, neurotic disorder, or personality disorder) and also non-clinical women [38]. Only 32 women with psychosis were included in the analysis because 11 (two of whom have DD) were excluded for being peri or postmenopausal. Findings were that 56% of the included patients were admitted to hospital during the low follicular phase of the menstrual cycle, which suggests that low estradiol levels were associated with an exacerbation of psychotic symptoms.

Focusing on the influence of reproductive variables on the clinical course of DD, González-Rodríguez and collaborators explored psychopathological symptoms in a cohort of 80 women with DD diagnosed using the Diagnostic and Statistical Manual for Mental Disorders, Fourth Edition, Text Revision (DSM-IV-TR) criteria [39]. Psychopathological symptoms were assessed by the Positive and Negative Syndrome Scale (PANSS) for psychotic symptoms, depressive symptoms were assessed by the 17-item Hamilton Rating Depression Scale (17-HRDS), and suicidality by the Columbia–Suicide Severity Rating Scale (C–SSRS). Fifty-seven women completed the trial. They were divided into two groups according to premenopausal and postmenopausal onset of symptoms. The women in the premenopausal onset group showed more erotomanic delusions and delusions with sexual content than those with postmenopausal onset. On the other hand, postmenopausal onset women more frequently presented with jealousy and somatic delusions.

The same research team investigated reproductive variables and use of gynecological services in a group of 25 female outpatients with DD [40]. Sociodemographic and clinical variables were recorded, as well as the following reproductive variables: age at menarche, age at menopause, use of contraceptives, menstrual patterns, gynecological disorders, and number of previous pregnancies and abortions. Utilization rates of gynecological services were also recorded. Mean age at onset was 48 years, mean age at menarche was 12.8 years, and mean age at menopause was 48.7. Persecutory delusions were most common in this sample, followed by erotomanic delusions. Age at onset of the disorder was not used as a variable.

**Table 2:** Putative association of sex/gender with delusional content in DD.

Potential Association	Main Findings	Reference
Negative	No differences in somatic, jealous and erotomanic delusions between men and women	Wustmann et al., 2011 [31] Kulkarni et al., 2017 [33] De Portugal et al., 2010 [34]
Positive	Erotomania more common in women than men	Román-Avezuela et al., 2015 [32]
Positive	Erotomania more likely in premenopausal than postmenopausal women	González-Rodríguez et al., 2015a [39]
Positive	Jealous and somatic delusions common in women with postmenopausal onset	González-Rodríguez et al., 2015a [39]

### 3.2. Potential Effects of Sex/Gender on Affective Comorbidity and Anxiety Disorders in DD

Psychiatric comorbid disorders (e.g., affective and anxiety disorders) are not rare in the context of DD. In general, affective symptoms are more frequent in women than in men. In DD, the prevalence of depressive disorders has been estimated at 21–55% [41].

Table 3 presents results of the association between sex/gender and affective comorbidity in DD.

De Portugal and collaborators carried out a cross-sectional study that included 86 outpatients with DD [34]. Sixty-two per cent of the sample were women and the mean age of the women was 55.1 (e.g., largely postmenopausal). The mean age of the men was 52.2. No statistically significant differences were found between women and men in the presence of depression, nor the severity of depressive symptoms as measured by the Montgomery–Asberg Depression Rating Scale (MADRS). The PANSS General Psychopathologic subscale was higher in men than in women. In other words, the men, though younger as a group, were more severely ill and their level of depression was equal to that of the largely postmenopausal women.

A similar study by González-Rodríguez and collaborators [39] investigated depressive symptoms in a cohort of postmenopausal women followed prospectively for 24 months and longer. The sample was divided into DD women with premenopausal and postmenopausal onset. After controlling for duration of untreated psychosis (DUP), antipsychotic dosage in chlorpromazine equivalent doses (CPZE), educational levels, and psychopathological baseline scores, women with onset in premenopause showed more depressive symptoms than those with postmenopausal onset. DD women in the perimenopausal period were not included. The same research team investigated the correlation between age at menarche, age at menopause, and psychopathology in a group of 25 female outpatients with DD [40]. Age at menopause was 48.7 years and age at menarche was 12.8 years. Neither variable was associated with psychotic or depressive symptoms.

In a tertiary care center in India, Kulkarni et al. [33] carried out a case register study by reviewing medical records of 455 patients with DD (48.1% women). Men and women were comparable in age. There were no gender differences in depressive symptoms. Leinonen and collaborators [36] followed patients with major depressive disorders and DD over 10 years and observed that psychogeriatric patients admitted to hospital for severe mental illness presented a high risk of organic dementia. In the subgroup of 18 patients with DD, the vast majority of whom were women, there was no specific mention of depressive symptoms.

Román-Avezuela and collaborators [32] retrospectively investigated cases of 50 inpatients with DSM-IV DD during their first psychiatric admission. The age of first admission was higher in women than in men (52.07 vs. 45). Women suffered more frequently from insomnia than men; however, no statistically significant differences in rates of depression were found between women and men.

**Table 3:** Putative sex/gender difference and affective comorbidity in DD.

Potential Association	Main Results	Reference
Negative	No difference in the presence or severity of depression between men and women with DD	Román-Avezuela et al., 2015 [32] Kulkarni et al., 2017 [33] De Portugal et al., 2010 [34]
Positive	More depression in women with premenopausal DD onset than with postmenopausal onset	González-Rodríguez et al., 2015 [39]
Negative	Ages at menarche and menopause were not associated with depressive symptoms in women with DD	González-Rodríguez et al., 2015b [40]

Abbreviations: DD, Delusional Disorder.

During the reproductive years, women experience not only depression but also anxiety symptoms at times of hormonal change (premenstrually, postpartum, perimenopause); at menopause, both anxiety and mood symptoms become more severe and occur more frequently than before [42,43].

De Portugal and collaborators evaluated 86 outpatients (33 m; 53 f) with DD who, using the Mini International Neuropsychiatry Interview (MINI), fulfilled DSM-IV criteria [44]. Almost half (46%) suffered from at least one additional psychiatric comorbidity. Anxiety disorders were diagnosed in eight patients (14%), most being women. The proportion of postmenopausal women was not reported. No differences were found between men and women in terms of functioning.

### 3.3. The Effects of Sex/Gender on Substance Use Disorders in Delusional Disorder

Many studies have reported that the rate of substance use disorder in the general population is higher in men than in women, but whether consequences distinguish men and women remains controversial [45,46].

In the context of DD, De Portugal and collaborators carried out a cross-sectional study investigating clinical features in 86 outpatients with this diagnosis [34]. A systematic inventory was used to register sociodemographic variables as well as clinical features. Premorbid substance use defined by DSM-IV criteria was also recorded. Men showed a significantly higher frequency of premorbid substance abuse than women (30.3% vs. 11.3%). No specific mention was made of the reproductive status in women participants. The higher frequency in men is in agreement with the findings of Román-Avezuela and collaborators who investigated clinical features in a sample of DD inpatients [32]. Men had more substance use disorders than women (40.9% vs. 3.6%). Cannabis abuse and dependence was more frequent (22.7% vs. 0%) in men, as was alcohol use disorder (22.7% vs. 3.6%) When analyzing substance use disorders by their onset prior or post DD, men were more likely to be diagnosed with substance use disorders at least one month before the DD diagnosis than were women (40.9% vs. 3.6%).

Along the same lines, Kulkarni et al. investigated sociodemographic and clinical characteristics in a sample of patients with DD from India [33]. The frequency of comorbid substance use disorders was significantly higher in men than in women (24.1% vs. 1.8%), which could explain the substantial occupational dysfunction found in men.

Delusional jealousy is frequently associated with neurological and psychiatric disorders [47], and alcohol use disorders. Kulkarni and collaborators [33] found that the false belief of partner’s infidelity was the most common delusion, particularly in men. The high frequency of substance use disorders in men may help to explain this finding, which is consistent with the results from a cross-sectional study in first-episode treatment-naïve psychosis patients recruited in a tertiary care center in northern India [48]. The sample included 13 delusional disorder participants. A modified semi-structured interview was used to record sociodemographic and clinical characteristics, including information with regard to the use of substances: starting age, type of substance, last intake of substance, duration of substance use, and pattern of use. Tetrahydrocannabinol urine concentrations were obtained by immune assay. The Alcohol Use Disorder Identification Test (AUDIT) was used to detect alcohol use. Once again, men in the total sample were more frequently diagnosed with alcohol use disorders than were women.

### 3.4. The Effect of Sex/Gender on Suicide Risk and Aggressivity in Delusional Disorder

In the general population, it is well known that women make more suicide attempts than men, but men’s attempts are much more often successful [49,50]. Men use more lethal means, but lethality is more common in men than women independently of the method used.

In the context of DD, González-Rodríguez and collaborators carried out a prospective observational study with a 24-month follow-up on consecutive cases of DD women attending an outpatient service [39]. Lifetime and follow-up suicidal ideation and suicidal behavior were assessed using the Columbia–Suicide Severity Rating Scale (C–SSRS). The sample was divided into two groups according to the reproductive status of the women at the time of DD onset, premenopause and postmenopause. There were no statistically significant group differences in terms of functioning, intensity of suicidal intention, or suicidal behavior. The timing of DD onset did not affect suicidality measures. The same research group carried out a case register study of 25 women with DD and found a positive correlation between age at menopause and the intensity of suicidal ideation: the older the age at menopause, the stronger the suicidal urges [40]. In an inpatient sample of 50 patients with DD, Román-Avezuela and collaborators [32] found no statistically significant differences in suicidal ideation between men and women (13.6% vs. 10.7%) [32].

De Portugal and collaborators investigated risk of aggressivity in a sample of 86 inpatients with DD [34]. No statistical gender differences were reported; however, men were more likely than women to present with an acute onset. In fact, very few studies have explored the risk of aggressivity in the DD population. Herbel and Stelmach [51] studied characteristics and behaviors of 22 DD prisoners but could not make gender comparisons because all 22 were men.

### 3.5. The Effect of Sex/Gender on Cognition in Delusional Disorder

Cognitive performance is considered to differ between men and women in the general population. In a study investigating cognitive functions in 21 male and 21 female students aged 19–37 years old, cognitive assessment was undertaken once in men and, in women, once during a preovulatory menstrual period and once in a postovulatory period [52]. A variety of cognitive functions were tested, and all results proved similar between men and women in their preovulatory cycle phase. During the postovulatory (high estrogen phase of the cycle), women showed advantages in the executive task (Stroop test) and disadvantages in voice response time, an attentional task. Few studies have specifically assessed neurocognitive performance in DD.

Grover and collaborators compared attention, concentration, executive functions, and memory in 20 patients with DD, as well as 20 patients with schizophrenia and 20 healthy controls [23]. Results were adjusted by taking sex, age, and level of education into account. Clinical stability of at least three months, defined by the absence of symptom exacerbation as reported by patients, relatives, or medical records review was required for participation. Dose of antipsychotic medications could not have been increased by more than 50% during those three months. The results showed that patients with DD had significantly more impairment of attention, visual learning and visual memory, verbal working memory, and executive functions, than patients with schizophrenia. No gender differences were reported.

De Portugal and colleagues found no statistically significant differences in cognitive measures between men and women with DD as measured by the Mini Mental State Examination [34].

## 4. Discussion

The aim of this review was to investigate the potential effects of sex/gender on the psychopathology (delusional themes, depressive and anxiety comorbidity, substance use disorders, risk of suicide and aggression, and cognition) of DD. In schizophrenia sex hormones are able to be studied directly in animal models, however this was not possible here because there are no animal models of delusional disorders and no human studies in which sex hormone levels have been assessed.

Several studies have reported that women with DD show an older age at onset of symptoms as well as age at first hospital admission than men, and their DD diagnosis is more stable, less inclined to change over time [31]. Specific delusional themes (e.g., erotomania and delusional parasitosis) have been anecdotally associated with women, but Wustmann et al. [31] found no gender differences in delusional themes, while Román-Avezuela et al. [32] reported more persecutory delusions in men and confirmed the higher rate of erotomania in women. Differences in delusional content between men and women, if they are confirmed to exist, would suggest a gender e.g., sociocultural effect rather than a biological sex effect. Kulkarni et al. [33] in a study from India, show how culture and tradition can affect delusional themes. Patient age, a biological effect, may also affect the content of delusional themes. Korner et al. [53] report that, in geriatric populations, the themes most frequently found center around persecution. This may be partially explained by the presence of incipient dementia since dementing disorders are closely associated with persecutory delusions [54].

The interest in male/female differences in depression and anxiety comorbidity in DD stems from the well-known fact that internalizing disorders (problems attributed to the self) are significantly more prevalent in women than in men [55,56,57]. These differences probably originate both from sex (an inherently more reactive stress reactivity in women) and from gender (socialization differences and trauma exposure differences between males and females found in many parts of the world). In most DD studies we reviewed, no sex/gender differences were found in the presence or severity of comorbid depression [32]. The lack of sex differences was consistent in samples of inpatients [32], outpatients, [34] and in mixed samples [33]. The conclusion could be that depression is such an integral part of DD that potential sex differences are obliterated or that the generally late mid-life onset of DD effaces the biological effects of sex hormone differences that are putatively responsible for depression. The latter explanation is consistent with the fact that women with premenopausal onset of DD do show more depressive symptoms than those with postmenopausal onset [39]. Another possibility is that common menopausal transition symptoms (insomnia, irritability, mood swings, and cognitive symptoms) [49] may overlap with depressive symptoms in premenopausal onset women. Age at menarche and age at menopause (indices of cumulative estrogen levels) did not correlate with the presence of depressive symptoms [40]. Rocca and collaborators [58] found long-term risk of depression and anxiety in women after bilateral oophorectomy. Thus, psychopathological effects of the loss of estrogens may differ according to the type of menopause: natural vs. surgical.

The vast majority of studies have reported a higher prevalence of substance use disorders in men suffering from DD compared to women [32,34]. Particularly, alcohol use disorders and the development of jealous delusions have been frequently found to be associated with men [38]. A higher frequency of substance use disorders prior to the onset of DD has been described in men with DD compared to women. This is probably a reflection of the relatively high substance use of men in the general population.

Suicide risk is tied to depression, but the male/female difference is probably more associated with gender than with sex. Women have more access to prescription medications than men and men have more access to guns, which means that suicidal men use more lethal means—women may thus attempt suicide more often, but men more often complete suicide [59]. Furthermore, women are more likely than men to report suicidal ideation and to, thus, receive protective social support [60,61]. However, age at menopause was positively associated with the intensity of suicidal ideation in sample of 25 women with DD [40], which suggests that neuroprotection conferred by estrogens may play a role. The onset of DD (premenopausal or postmenopausal) did not have an impact on suicidality in women with DD [39]. A recent review revealed that both individual and community level factors affect suicidal ideation in postmenopausal women [53]. In the particular context of DD, psychosocial risk factors have also been found to be associated with higher rates of DD in middle- and working-class neighborhoods than elsewhere [62].

Few studies have investigated gender differences in the risk of aggressivity in patients with DD, but men more often than women present for care in an acute state [34] and may, thus, be perceived to be more aggressive.

The pattern of cognitive function in patients with DD remains unclear. Some authors have tried to investigate cognitive performance in people with DD and compare them with those found in schizophrenia populations [63]. A recent study on the topic revealed that verbal memory and other cognitive symptoms were impaired in DD, and these were related to poor functionality.

The studies we cite considered the potential effects of depression or anxiety comorbidity as well as substance abuse and, most importantly, the menopausal state, on sex/gender difference. What they did not consider were individual genetic differences and group genetic differences between men and women. There may exist DD-associated genes that are sex-biased and that account for a substantial portion of male/female difference. This has, thus far, not been studied.

This review is limited by the paucity of relevant studies. To date, research in DD has mainly been based on observational studies, case series, and case reports. The reported findings are, therefore, tentative. A further limitation is that, though most studies report excluding organic psychoses during recruitment, they do not report what assessment tools they used to make these exclusions. As to the representativeness of our literature search, we realize that all the papers we cite were written in English although we scanned for three other languages known to the authors. Ideally, we would have wanted to review literature from around the world because it is important to know whether male/female are products of biology or of gender, or both [64].

To the best of our knowledge, however, this is the first review investigating the potential association of sex/gender and, by implication, sex hormones with clinical features of DD. It is hoped that future studies will recruit large samples and be able to directly measure levels of gonadal hormones because this could lead to improvements in treatment.

## 5. Conclusions

Sex and gender difference in the epidemiology, clinical expression, treatment, and outcome of psychiatric disorders is a topic of great interest and controversy. The controversy often centers around the impact of biological versus sociocultural explanations for difference, but very little of this work has been done in the field of delusional disorders. We have reviewed the sparse literature that exists and conclude that differences between men and women are relatively few, but that some differences point to the influence of menopause in symptomatic expression and comorbidity. As this field of research expands, it may lead to more individualized and, thus, more effective treatments.

## Figures and Tables

**Table 1:** Scores of the Scale for the Assessment of Narrative Review (SANRA).

Item 1	Item 2	Item 3	Item 4	Item 5	Item 6	Total Score
Justification of the article’s importance	Aims and formulation of questions	Description of literature search	Referencing	Scientific reasoning	Presentation of data	Sum of scores
The importance is explicitly justified (2)	One or more concrete aims or questions are formulated (2)	The literature search is described briefly (1)	Key statements are supported by references (2)	Appropriate evidence is present (adequately described) (2)	Relevant outcome data are generally presented appropriately (2)	11

## Data Availability

The data presented in this review are available upon request from the corresponding author.
